# Diethyl 4-(2-meth­oxy­phen­yl)-2,6-di­methyl-1,4-di­hydro­pyridine-3,5-di­carboxyl­ate

**DOI:** 10.1107/S1600536813009951

**Published:** 2013-04-24

**Authors:** Ke Wang, Yifeng Wang, Minjie Yao, Danqian Xu

**Affiliations:** a Catalytic Hydrogenation Research Center, Zhejiang University of Technology, Hangzhou 310014, People’s Republic of China

## Abstract

In the title compound, C_20_H_25_NO_5_, the di­hydro­pyridine ring adopts a flattened boat conformation. The meth­oxy­phenyl ring is almost perpendicular to the mean plane of the pyridine ring [dihedral angle = 88.42 (3)°]. The two carbonyl units adopt a synperiplanar conformation with respect to the double bonds in the di­hydro­pyridine ring. In the crystal, mol­ecules are connected by N—H⋯O hydrogen bonds into *R*_4_^4^(24) tetra­meric rings. A region of disordered electron density, located at the center of four adjacent mol­ecules, was treated with the SQUEEZE routine in *PLATON* [Spek (2009 ▶). *Acta Cryst.* D**65**, 148–155]. It is probably the result of traces of the solvent of crystallization and was not taken into account during the structure refinement.

## Related literature

For general background to 1,4-di­hydro­pyridine compounds, see: Franke *et al.* (2008 ▶); Takemoto *et al.* (2010 ▶). For related structures, see: Fun *et al.* (2012 ▶); Kapoor *et al.* (2011 ▶).
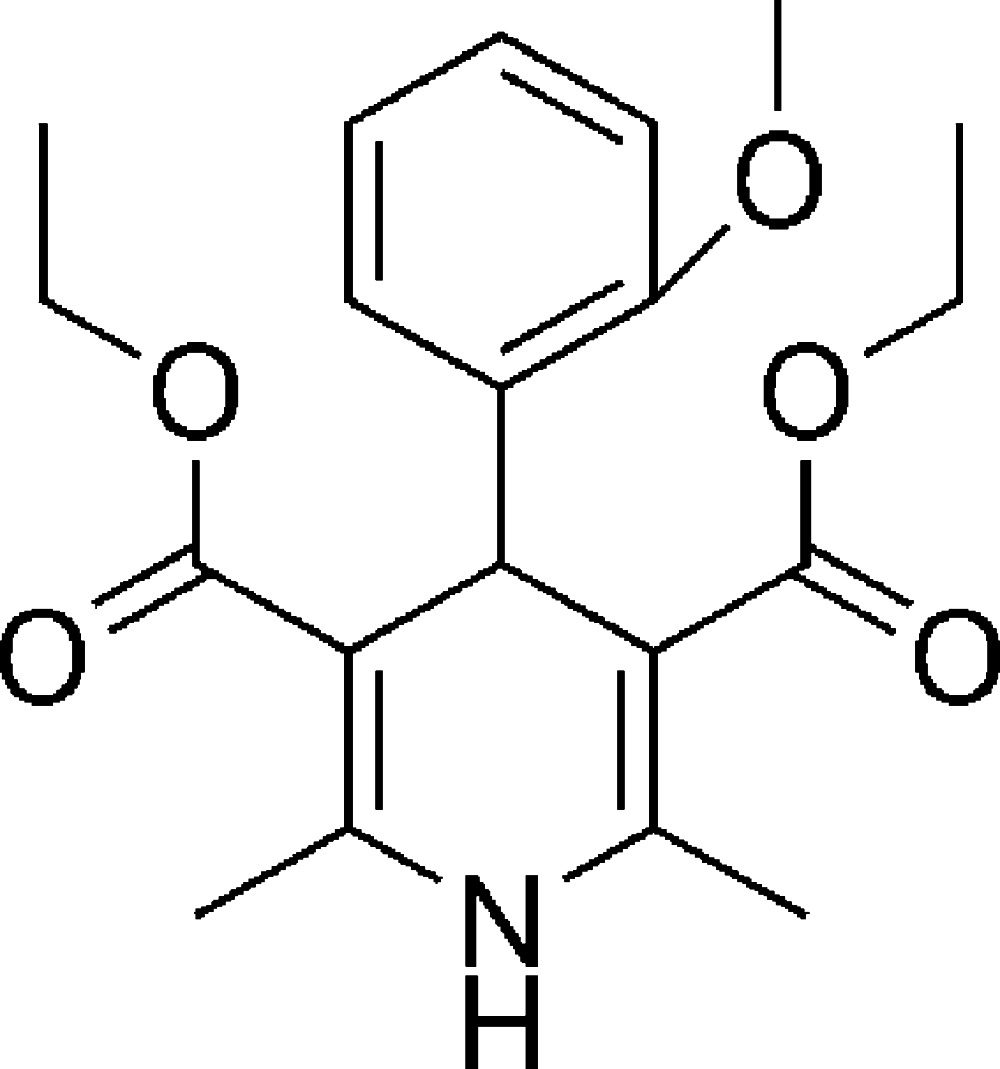


## Experimental

### 

#### Crystal data

C_20_H_25_NO_5_
*M* *_r_* = 359.41
Tetragonal, 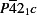

*a* = 22.4689 (7) Å
*c* = 8.2443 (3) Å
*V* = 4162.1 (2) Å^3^
*Z* = 8
Mo *K*α radiation
μ = 0.08 mm^−1^
*T* = 296 K
0.47 × 0.31 × 0.22 mm

#### Data collection

Rigaku R-AXIS RAPID/ZJUG diffractometer
Absorption correction: multi-scan (*ABSCOR*; Higashi, 1995 ▶) *T*_min_ = 0.952, *T*_max_ = 0.982
60800 measured reflections
4755 independent reflections
3128 reflections with *I* > 2σ(*I*)
*R*_int_ = 0.078

#### Refinement

*R*[*F* ^2^ > 2σ(*F* ^2^)] = 0.059
*wR*(*F* ^2^) = 0.167
*S* = 1.00
4755 reflections
241 parameters
H-atom parameters constrained
Δρ_max_ = 0.17 e Å^−3^
Δρ_min_ = −0.20 e Å^−3^

### 

Data collection: *PROCESS-AUTO* (Rigaku, 2006 ▶); cell refinement: *PROCESS-AUTO*; data reduction: *CrystalStructure* (Rigaku, 2007 ▶); program(s) used to solve structure: *SHELXS97* (Sheldrick, 2008 ▶); program(s) used to refine structure: *SHELXL97* (Sheldrick, 2008 ▶); molecular graphics: *ORTEP-3 for Windows* (Farrugia, 2012 ▶); software used to prepare material for publication: *WinGX* (Farrugia, 2102).

## Supplementary Material

Crystal structure: contains datablock(s) global, I. DOI: 10.1107/S1600536813009951/pk2475sup1.cif

Structure factors: contains datablock(s) I. DOI: 10.1107/S1600536813009951/pk2475Isup2.hkl

Supplementary material file. DOI: 10.1107/S1600536813009951/pk2475Isup3.cml

Additional supplementary materials:  crystallographic information; 3D view; checkCIF report

## Figures and Tables

**Table 1:** Hydrogen-bond geometry (Å, °)

*D*—H⋯*A*	*D*—H	H⋯*A*	*D*⋯*A*	*D*—H⋯*A*
N1—H1*A*⋯O5^i^	0.86	2.07	2.924 (3)	170

Symmetry code: (i) 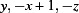.

**Fig. 1.**
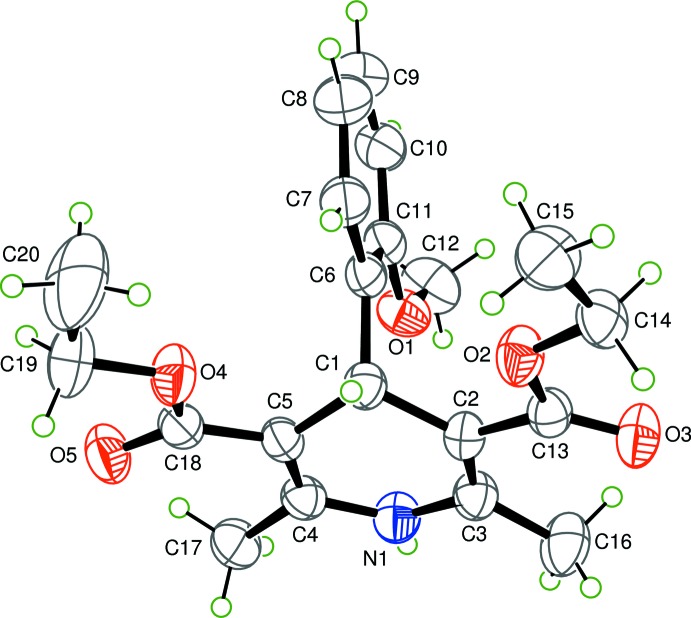
The structure of the title compound. Displacement ellipsoids are drawn at the 40% probability level.

**Fig. 2.**
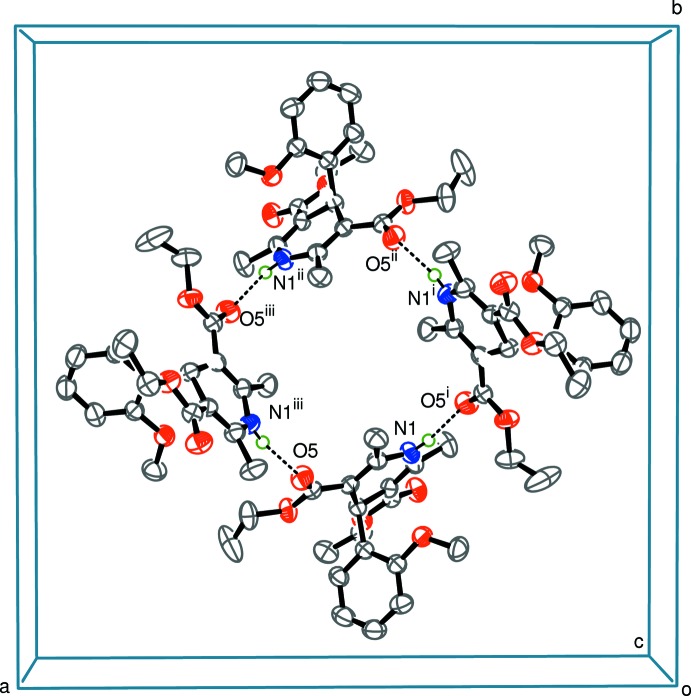
The molecular packing of the title compound showing the R44(24) tetrameric rings, with N1—H1···O5 hydrogen bonds shown as dashed lines.

## supplementary crystallographic information

### Comment

1,4-Dihydropyridine compounds are important drugs by virtue of their pharmacological activities, and they are used in the treatment of a number of diseases, such as cardiovascular diseases and Alzheimer's disease. In this article, the crystal structure of the dihydropyridine compoud diethyl 4-(2-methoxyphenyl)-2,6- dimethyl-1,4-dihydropyridine-3,5-dicarboxylate is described (Fig. 1). The dihydropyridine ring adopts a flattened boat conformation. The methoxyphenyl ring is almost perpendicular to the mean plane of the pyridine ring [dihedral angle = 88.42 (3)°]. The two carbonyl units adopt a synperiplanar conformation with respect to the double bonds in the dihydropyridine ring. In the crystal, the molecules are connected by intermolecular N—H···O hydrogen bonds into *R*_4_^4^(24) tetrameric rings. A region of disordered electron density, located at the center of four adjacent molecules, was treated using the SQUEEZE routine in *PLATON* (Spek, 2009), which indicated a solvent-accessible void of of 178 Å^3^. It is probably due to traces of the solvent of crystallization and was not taken into account during structure refinement.

### Experimental

The mixture of 2-methoxybenzaldehyde (1 mmol), ethylacetoacetate (2 mmol) and ammonium acetate(1 mmol) was stirred at at 343 K for 3 h (monitored by TLC). Then the mixture was purified by flash column chromatography (silica gel, Hex/AcOEt, *v*/*v*, 3:1) giving the title compound. Single crystals were obtained by slow evaporation of a CH_2_Cl_2_ and n-Hexane solution.

### Refinement

A region of disordered electron density, located at the center of four adjacent molecules, was treated using the SQUEEZE routine in *PLATON* (Spek, 2009). It gave a solvent-accessible void of of 178 Å^3^. It is probably due to traces of the solvent of crystallization and was not taken into account during structure refinement. H atoms were placed in calculated positions and treated as riding atoms: N—H = 0.86 Å, C—H = 0.98 Å (*sp*), C—H = 0.97 Å (sp2), C—H = 0.96 Å (sp3) and C—H = 0.93 Å (aromatic) with *U*_iso_(H) = 1.2*U*_eq_ (N, C).

### Crystal data

**Table:**

C_20_H_25_NO_5_	*D*_x_ = 1.147 Mg m^−^^3^
*M**_r_* = 359.41	Mo *K*α radiation, λ = 0.71073 Å
Tetragonal, *P*42_1_*c*	Cell parameters from 36625 reflections
Hall symbol: P -4 2n	θ = 3.1–27.4°
*a* = 22.4689 (7) Å	µ = 0.08 mm^−^^1^
*c* = 8.2443 (3) Å	*T* = 296 K
*V* = 4162.1 (2) Å^3^	Block, colorless
*Z* = 8	0.47 × 0.31 × 0.22 mm
*F*(000) = 1536	

### Data collection

**Table:**

Rigaku R-AXIS RAPID/ZJUG diffractometer	4755 independent reflections
Radiation source: rotating anode	3128 reflections with *I* > 2σ(*I*)
Graphite monochromator	*R*_int_ = 0.078
Detector resolution: 10.00 pixels mm^-1^	θ_max_ = 27.5°, θ_min_ = 3.1°
ω scans	*h* = −20→20
Absorption correction: multi-scan (*ABSCOR*; Higashi, 1995)	*k* = 0→29
*T*_min_ = 0.952, *T*_max_ = 0.982	*l* = 0→10
60800 measured reflections	

### Refinement

**Table:**

Refinement on *F*^2^	Primary atom site location: structure-invariant direct methods
Least-squares matrix: full	Secondary atom site location: difference Fourier map
*R*[*F*^2^ > 2σ(*F*^2^)] = 0.059	Hydrogen site location: inferred from neighbouring sites
*wR*(*F*^2^) = 0.167	H-atom parameters constrained
*S* = 1.00	*w* = 1/[σ^2^(*F*_o_^2^) + (0.0855*P*)^2^ + 0.9222*P*] where *P* = (*F*_o_^2^ + 2*F*_c_^2^)/3
4755 reflections	(Δ/σ)_max_ = 0.008
241 parameters	Δρ_max_ = 0.17 e Å^−^^3^
0 restraints	Δρ_min_ = −0.20 e Å^−^^3^

### Special details

**Table:**

Geometry. All e.s.d.'s (except the e.s.d. in the dihedral angle between two l.s. planes) are estimated using the full covariance matrix. The cell e.s.d.'s are taken into account individually in the estimation of e.s.d.'s in distances, angles and torsion angles; correlations between e.s.d.'s in cell parameters are only used when they are defined by crystal symmetry. An approximate (isotropic) treatment of cell e.s.d.'s is used for estimating e.s.d.'s involving l.s. planes.
Refinement. Refinement of *F*^2^ against ALL reflections. The weighted *R*-factor *wR* and goodness of fit *S* are based on *F*^2^, conventional *R*-factors *R* are based on *F*, with *F* set to zero for negative *F*^2^. The threshold expression of *F*^2^ > σ(*F*^2^) is used only for calculating *R*-factors(gt) *etc*. and is not relevant to the choice of reflections for refinement. *R*-factors based on *F*^2^ are statistically about twice as large as those based on *F*, and *R*- factors based on ALL data will be even larger.

### Fractional atomic coordinates and isotropic or equivalent isotropic displacement parameters (Å^2^)

**Table:**

	*x*	*y*	*z*	*U*_iso_*/*U*_eq_	
C1	0.48258 (11)	0.26476 (11)	0.3185 (3)	0.0488 (6)	
H1	0.5183	0.2658	0.3866	0.059*	
C2	0.43244 (11)	0.29128 (12)	0.4187 (3)	0.0487 (6)	
C3	0.39385 (13)	0.33091 (13)	0.3545 (4)	0.0559 (7)	
C4	0.45445 (13)	0.34162 (12)	0.1122 (3)	0.0524 (7)	
C5	0.49582 (12)	0.30287 (11)	0.1698 (3)	0.0482 (6)	
C6	0.47208 (12)	0.19936 (12)	0.2738 (3)	0.0485 (6)	
C7	0.51178 (14)	0.15615 (13)	0.3240 (4)	0.0623 (8)	
H7	0.5442	0.1673	0.3872	0.075*	
C8	0.50466 (18)	0.09635 (15)	0.2830 (5)	0.0797 (10)	
H8	0.5316	0.0681	0.3207	0.096*	
C9	0.45815 (16)	0.07942 (15)	0.1875 (5)	0.0802 (10)	
H9	0.4540	0.0397	0.1575	0.096*	
C10	0.41693 (16)	0.12109 (14)	0.1349 (4)	0.0705 (8)	
H10	0.3851	0.1095	0.0700	0.085*	
C11	0.42368 (13)	0.18025 (12)	0.1801 (4)	0.0546 (7)	
C12	0.33177 (15)	0.20776 (18)	0.0506 (5)	0.0826 (11)	
H12A	0.3415	0.1878	−0.0490	0.124*	
H12B	0.3088	0.2428	0.0273	0.124*	
H12C	0.3090	0.1815	0.1184	0.124*	
C13	0.42649 (12)	0.27102 (13)	0.5869 (3)	0.0515 (6)	
C14	0.47214 (15)	0.21103 (14)	0.7908 (3)	0.0628 (8)	
H14A	0.4387	0.1840	0.8019	0.075*	
H14B	0.4685	0.2420	0.8722	0.075*	
C15	0.52918 (17)	0.17829 (16)	0.8121 (5)	0.0817 (10)	
H15A	0.5305	0.1453	0.7383	0.123*	
H15B	0.5319	0.1639	0.9215	0.123*	
H15C	0.5619	0.2046	0.7904	0.123*	
C16	0.34108 (15)	0.35877 (18)	0.4346 (5)	0.0817 (10)	
H16A	0.3139	0.3282	0.4683	0.123*	
H16B	0.3215	0.3849	0.3594	0.123*	
H16C	0.3539	0.3811	0.5275	0.123*	
C17	0.45761 (16)	0.37777 (15)	−0.0409 (4)	0.0698 (9)	
H17A	0.4739	0.4163	−0.0171	0.105*	
H17B	0.4184	0.3823	−0.0853	0.105*	
H17C	0.4826	0.3579	−0.1182	0.105*	
C18	0.55359 (12)	0.29701 (12)	0.0932 (4)	0.0525 (7)	
C19	0.65098 (15)	0.2576 (2)	0.1250 (5)	0.0846 (11)	
H19A	0.6722	0.2949	0.1383	0.102*	
H19B	0.6512	0.2471	0.0108	0.102*	
C20	0.6793 (2)	0.2111 (3)	0.2197 (7)	0.140 (2)	
H20A	0.6677	0.2149	0.3313	0.210*	
H20B	0.7218	0.2148	0.2110	0.210*	
H20C	0.6672	0.1729	0.1794	0.210*	
N1	0.40319 (10)	0.35131 (11)	0.1988 (3)	0.0599 (6)	
H1A	0.3752	0.3713	0.1532	0.072*	
O1	0.38505 (9)	0.22427 (9)	0.1319 (3)	0.0636 (6)	
O2	0.47297 (9)	0.23683 (10)	0.6302 (2)	0.0633 (6)	
O3	0.38594 (9)	0.28131 (11)	0.6811 (3)	0.0712 (6)	
O4	0.59075 (8)	0.26366 (10)	0.1817 (3)	0.0654 (6)	
O5	0.56955 (10)	0.31897 (11)	−0.0357 (3)	0.0738 (6)	

### Atomic displacement parameters (Å^2^)

**Table:**

	*U*^11^	*U*^22^	*U*^33^	*U*^12^	*U*^13^	*U*^23^
C1	0.0473 (14)	0.0528 (14)	0.0462 (13)	0.0037 (11)	0.0026 (12)	0.0049 (12)
C2	0.0461 (14)	0.0570 (15)	0.0429 (13)	−0.0004 (11)	0.0039 (11)	−0.0004 (12)
C3	0.0520 (15)	0.0602 (17)	0.0554 (16)	0.0044 (13)	0.0034 (13)	0.0039 (13)
C4	0.0585 (16)	0.0496 (14)	0.0490 (15)	−0.0008 (12)	−0.0007 (13)	0.0047 (12)
C5	0.0511 (14)	0.0492 (14)	0.0442 (14)	−0.0067 (12)	0.0027 (12)	0.0024 (11)
C6	0.0525 (14)	0.0499 (14)	0.0432 (13)	0.0026 (11)	0.0073 (12)	0.0077 (11)
C7	0.0605 (17)	0.0605 (17)	0.0659 (18)	0.0080 (14)	0.0056 (15)	0.0099 (15)
C8	0.086 (2)	0.0608 (19)	0.092 (3)	0.0170 (17)	0.011 (2)	0.0100 (18)
C9	0.089 (2)	0.0545 (18)	0.097 (3)	0.0006 (17)	0.012 (2)	0.0019 (19)
C10	0.081 (2)	0.0586 (18)	0.072 (2)	−0.0094 (16)	0.0081 (17)	−0.0001 (16)
C11	0.0564 (15)	0.0543 (15)	0.0530 (15)	−0.0003 (13)	0.0088 (13)	0.0092 (13)
C12	0.063 (2)	0.088 (2)	0.096 (3)	−0.0112 (18)	−0.0207 (18)	0.013 (2)
C13	0.0530 (15)	0.0578 (15)	0.0439 (14)	−0.0028 (13)	0.0014 (12)	−0.0015 (12)
C14	0.0764 (19)	0.0706 (18)	0.0415 (14)	−0.0027 (15)	0.0005 (14)	0.0091 (14)
C15	0.102 (3)	0.080 (2)	0.063 (2)	0.017 (2)	−0.004 (2)	0.0156 (18)
C16	0.0598 (19)	0.102 (3)	0.084 (2)	0.0253 (19)	0.0137 (18)	0.006 (2)
C17	0.083 (2)	0.0630 (18)	0.0630 (19)	0.0057 (17)	0.0066 (17)	0.0187 (16)
C18	0.0548 (15)	0.0510 (14)	0.0516 (15)	−0.0058 (12)	0.0025 (13)	0.0010 (13)
C19	0.0505 (17)	0.123 (3)	0.080 (2)	0.0072 (19)	0.0162 (17)	−0.003 (2)
C20	0.088 (3)	0.194 (6)	0.139 (4)	0.073 (3)	0.026 (3)	0.045 (4)
N1	0.0559 (14)	0.0657 (15)	0.0580 (15)	0.0110 (11)	0.0007 (12)	0.0094 (12)
O1	0.0616 (12)	0.0581 (12)	0.0711 (13)	−0.0028 (9)	−0.0114 (10)	0.0088 (10)
O2	0.0647 (12)	0.0804 (14)	0.0447 (10)	0.0107 (11)	0.0054 (9)	0.0094 (10)
O3	0.0668 (13)	0.0950 (16)	0.0517 (12)	0.0103 (12)	0.0162 (11)	0.0039 (11)
O4	0.0472 (10)	0.0853 (14)	0.0637 (13)	0.0086 (10)	0.0097 (10)	0.0092 (11)
O5	0.0707 (14)	0.0829 (15)	0.0678 (14)	−0.0057 (12)	0.0180 (11)	0.0189 (12)

### Geometric parameters (Å, º)

**Table:**

C1—C2	1.519 (4)	C12—H12C	0.9600
C1—C5	1.525 (4)	C13—O3	1.220 (3)
C1—C6	1.533 (4)	C13—O2	1.345 (3)
C1—H1	0.9800	C14—O2	1.446 (3)
C2—C3	1.351 (4)	C14—C15	1.488 (5)
C2—C13	1.466 (4)	C14—H14A	0.9700
C3—N1	1.379 (4)	C14—H14B	0.9700
C3—C16	1.494 (4)	C15—H15A	0.9600
C4—C5	1.359 (4)	C15—H15B	0.9600
C4—N1	1.373 (4)	C15—H15C	0.9600
C4—C17	1.503 (4)	C16—H16A	0.9600
C5—C18	1.449 (4)	C16—H16B	0.9600
C6—C7	1.382 (4)	C16—H16C	0.9600
C6—C11	1.401 (4)	C17—H17A	0.9600
C7—C8	1.395 (5)	C17—H17B	0.9600
C7—H7	0.9300	C17—H17C	0.9600
C8—C9	1.363 (5)	C18—O5	1.225 (3)
C8—H8	0.9300	C18—O4	1.338 (3)
C9—C10	1.387 (5)	C19—O4	1.438 (4)
C9—H9	0.9300	C19—C20	1.451 (6)
C10—C11	1.389 (4)	C19—H19A	0.9700
C10—H10	0.9300	C19—H19B	0.9700
C11—O1	1.374 (3)	C20—H20A	0.9600
C12—O1	1.422 (4)	C20—H20B	0.9600
C12—H12A	0.9600	C20—H20C	0.9600
C12—H12B	0.9600	N1—H1A	0.8600
			
C2—C1—C5	111.2 (2)	O2—C14—C15	107.2 (3)
C2—C1—C6	113.1 (2)	O2—C14—H14A	110.3
C5—C1—C6	112.0 (2)	C15—C14—H14A	110.3
C2—C1—H1	106.7	O2—C14—H14B	110.3
C5—C1—H1	106.7	C15—C14—H14B	110.3
C6—C1—H1	106.7	H14A—C14—H14B	108.5
C3—C2—C13	121.1 (2)	C14—C15—H15A	109.5
C3—C2—C1	121.5 (2)	C14—C15—H15B	109.5
C13—C2—C1	117.4 (2)	H15A—C15—H15B	109.5
C2—C3—N1	119.1 (3)	C14—C15—H15C	109.5
C2—C3—C16	127.8 (3)	H15A—C15—H15C	109.5
N1—C3—C16	113.1 (3)	H15B—C15—H15C	109.5
C5—C4—N1	119.6 (2)	C3—C16—H16A	109.5
C5—C4—C17	127.4 (3)	C3—C16—H16B	109.5
N1—C4—C17	113.0 (2)	H16A—C16—H16B	109.5
C4—C5—C18	121.2 (2)	C3—C16—H16C	109.5
C4—C5—C1	120.5 (2)	H16A—C16—H16C	109.5
C18—C5—C1	118.3 (2)	H16B—C16—H16C	109.5
C7—C6—C11	116.8 (3)	C4—C17—H17A	109.5
C7—C6—C1	120.1 (3)	C4—C17—H17B	109.5
C11—C6—C1	123.1 (2)	H17A—C17—H17B	109.5
C6—C7—C8	122.0 (3)	C4—C17—H17C	109.5
C6—C7—H7	119.0	H17A—C17—H17C	109.5
C8—C7—H7	119.0	H17B—C17—H17C	109.5
C9—C8—C7	119.8 (3)	O5—C18—O4	121.1 (3)
C9—C8—H8	120.1	O5—C18—C5	127.1 (3)
C7—C8—H8	120.1	O4—C18—C5	111.8 (2)
C8—C9—C10	120.3 (3)	O4—C19—C20	107.8 (3)
C8—C9—H9	119.9	O4—C19—H19A	110.1
C10—C9—H9	119.9	C20—C19—H19A	110.1
C9—C10—C11	119.3 (3)	O4—C19—H19B	110.1
C9—C10—H10	120.3	C20—C19—H19B	110.1
C11—C10—H10	120.3	H19A—C19—H19B	108.5
O1—C11—C10	122.9 (3)	C19—C20—H20A	109.5
O1—C11—C6	115.4 (2)	C19—C20—H20B	109.5
C10—C11—C6	121.7 (3)	H20A—C20—H20B	109.5
O1—C12—H12A	109.5	C19—C20—H20C	109.5
O1—C12—H12B	109.5	H20A—C20—H20C	109.5
H12A—C12—H12B	109.5	H20B—C20—H20C	109.5
O1—C12—H12C	109.5	C4—N1—C3	124.0 (2)
H12A—C12—H12C	109.5	C4—N1—H1A	118.0
H12B—C12—H12C	109.5	C3—N1—H1A	118.0
O3—C13—O2	121.3 (3)	C11—O1—C12	118.7 (3)
O3—C13—C2	127.7 (3)	C13—O2—C14	117.5 (2)
O2—C13—C2	111.0 (2)	C18—O4—C19	117.5 (3)
			
C5—C1—C2—C3	19.7 (4)	C9—C10—C11—C6	1.9 (5)
C6—C1—C2—C3	−107.3 (3)	C7—C6—C11—O1	−180.0 (2)
C5—C1—C2—C13	−161.9 (2)	C1—C6—C11—O1	−0.9 (4)
C6—C1—C2—C13	71.1 (3)	C7—C6—C11—C10	−2.2 (4)
C13—C2—C3—N1	176.3 (3)	C1—C6—C11—C10	176.8 (3)
C1—C2—C3—N1	−5.4 (4)	C3—C2—C13—O3	7.5 (5)
C13—C2—C3—C16	−1.2 (5)	C1—C2—C13—O3	−171.0 (3)
C1—C2—C3—C16	177.1 (3)	C3—C2—C13—O2	−173.4 (3)
N1—C4—C5—C18	−172.1 (2)	C1—C2—C13—O2	8.2 (3)
C17—C4—C5—C18	7.1 (4)	C4—C5—C18—O5	−9.0 (4)
N1—C4—C5—C1	7.3 (4)	C1—C5—C18—O5	171.6 (3)
C17—C4—C5—C1	−173.4 (3)	C4—C5—C18—O4	170.5 (3)
C2—C1—C5—C4	−20.6 (3)	C1—C5—C18—O4	−8.9 (3)
C6—C1—C5—C4	107.0 (3)	C5—C4—N1—C3	9.9 (4)
C2—C1—C5—C18	158.8 (2)	C17—C4—N1—C3	−169.4 (3)
C6—C1—C5—C18	−73.5 (3)	C2—C3—N1—C4	−11.0 (4)
C2—C1—C6—C7	−119.7 (3)	C16—C3—N1—C4	166.8 (3)
C5—C1—C6—C7	113.7 (3)	C10—C11—O1—C12	8.5 (4)
C2—C1—C6—C11	61.3 (3)	C6—C11—O1—C12	−173.8 (3)
C5—C1—C6—C11	−65.3 (3)	O3—C13—O2—C14	2.1 (4)
C11—C6—C7—C8	0.5 (4)	C2—C13—O2—C14	−177.1 (2)
C1—C6—C7—C8	−178.5 (3)	C15—C14—O2—C13	−177.1 (3)
C6—C7—C8—C9	1.5 (5)	O5—C18—O4—C19	3.7 (4)
C7—C8—C9—C10	−1.9 (6)	C5—C18—O4—C19	−175.9 (3)
C8—C9—C10—C11	0.2 (5)	C20—C19—O4—C18	−169.0 (4)
C9—C10—C11—O1	179.5 (3)		

### Hydrogen-bond geometry (Å, º)

**Table:**

*D*—H···*A*	*D*—H	H···*A*	*D*···*A*	*D*—H···*A*
N1—H1*A*···O5^i^	0.86	2.07	2.924 (3)	170

Symmetry code: (i) *y*, −*x*+1, −*z*.
